# High Health Care Utilization Preceding Diagnosis With Juvenile Idiopathic Arthritis

**DOI:** 10.1002/acr.80037

**Published:** 2026-04-22

**Authors:** Anna Costello, Rui Xiao, Xuemei Zhang, Jahan Jazayeri, Irit R. Rasooly, Pamela F. Weiss

**Affiliations:** ^1^ The Children's Hospital of Philadelphia Philadelphia Pennsylvania; ^2^ The Perelman School of Medicine at the University of Pennsylvania Philadelphia

## Abstract

**Objective:**

Although early diagnosis improves long‐term outcomes, patients with juvenile idiopathic arthritis (JIA) often experience prolonged, circuitous paths to diagnosis. To inform diagnostic improvement, we sought to characterize health care utilization in the year preceding diagnosis.

**Methods:**

We identified 10,021 patients with an incident diagnosis of JIA and 20,042 age‐ and sex‐matched healthy controls in the Merative MarketScan administrative datasets (2014–2022). Using negative binomial or hurdle models, we calculated incidence rate ratios (IRRs) comparing outpatient, inpatient, and emergency department or urgent care (ED/UC) utilization between patients with JIA and controls.

**Results:**

In the year before diagnosis, patients with JIA had significantly increased health care utilization compared to controls (IRR 2.55 [95% confidence interval (CI) 2.49–2.62], *P* < 0.001). Accounting for sex, age, and insurance, utilization was increased across care settings: outpatient (IRR 2.56 [95% CI 2.49–2.62], *P* < 0.001), inpatient (IRR 2.00 [95% CI 1.48–2.71], *P* < 0.001), and ED/UC encounters (IRR 1.76 [95% CI 1.67–1.86], *P* < 0.001). The most common visits by patients with JIA preceding diagnosis were to a general practitioner (91.8%), ED/UC (47.0%), and orthopedist (22.9%). Health care utilization increased as the index date approached. In patients insured by Medicaid, ED/UC care was more frequent (odds ratio 2.05 [95% CI 1.89–2.23]) and orthopedic care less frequent (odds ratio 0.27 [95% CI 0.24–0.34]) than in patients with commercial insurance.

**Conclusion:**

In the year before diagnosis, children with JIA have significantly higher health care utilization compared to healthy peers. There are differences in the patterns of utilization in patients with Medicaid versus commercial insurance. There may be opportunities for earlier identification of JIA in primary care, orthopedics, and ED/UC.

## INTRODUCTION

Juvenile idiopathic arthritis (JIA) can cause destructive joint damage, blindness from uveitis, and long‐lasting pain and disability.^1^ However, there is a growing therapeutic arsenal for managing JIA, including numerous safe medications that can halt disease progression.^2^ These medications are most effective when started early in the disease course,^3^ and early diagnosis and therapy have been shown to improve long‐term outcomes and increase the likelihood of preserved remission.^4^, ^5^, ^6^, ^7^ As such, timely recognition of the condition and timely initiation of rheumatologic care are crucial to enabling optimal outcomes for patients with JIA.

**SIGNIFICANCE & INNOVATIONS**

- Patients with juvenile idiopathic arthritis (JIA) have high health care utilization for at least a year preceding diagnosis, suggesting missed opportunities for an earlier diagnosis.
- Patients with JIA most often seek care from primary care physicians, emergency departments or urgent care centers, and orthopedic providers preceding diagnosis.
- There are differential health care utilization patterns between patients with Medicaid and those with commercial insurance, with increased utilization of emergency departments or urgent care centers and lower utilization of orthopedic care among patients with Medicaid.

Despite this diagnostic imperative, in practice, it is common to diagnose JIA in a patient who already has irreversible complications of longstanding disease, such as ocular scarring, leg length discrepancy, erosive joint damage, micrognathia, and joint contractures.^8^ Characterizing the health care utilization patterns that precede diagnosis is a critical step in identifying opportunities for earlier recognition in the diagnostic process and, ultimately, designing interventions to improve timely diagnosis. The existing literature is largely derived from international cohorts and utilizes retrospective chart review or prospective single‐center or multicenter surveys to capture health care utilization patterns.^9^, ^10^, ^11^, ^12^, ^13^, ^14^, ^15^, ^16^ These data are limited in their generalizability to US health care and its ability to account for care delivered across health care systems and settings. Thus, our study aim was to describe and quantify the health care utilization of children with JIA from the United States in the year preceding diagnosis, compared to a population of healthy controls, in a large, population‐based administrative dataset that includes patients with both commercial insurance and Medicaid.

## MATERIALS AND METHODS

### Study design

Retrospective cohort study comparing the health care utilization of children with an incident diagnosis of JIA to age‐ and sex‐matched health controls.

### Ethics

This study was deemed exempt from review by the Children's Hospital of Philadelphia Institutional Review Board (IRB 24‐022056).

### Data source

This study utilized deidentified data from the Merative MarketScan commercial and Medicaid databases from 2014 to 2022.^17^ The Merative MarketScan databases contain de‐identified patient‐level data, including diagnoses (*International Classification of Diseases, Ninth Revision* and *International Statistical Classification of Diseases and Related Health Problems, Tenth Revision* codes), inpatient and outpatient encounters, and pharmacy data (outpatient national drug codes, days’ supply, strength, and route of administration). The commercial and Medicaid databases are distinct patient samples, with the commercial database containing data for dependents covered by private health insurance plans throughout the United States and the Medicaid database presenting data for Medicaid enrollees from select states. Certain variables differ in availability, with race and ethnicity recorded only in the Medicaid dataset and geographic region recorded only in the commercial dataset.

### Participants

We identified patients ages 1 to 18 years with an incident diagnosis of JIA. Patients diagnosed between 16 and 18 years old were included in the study per the provisional Pediatric Rheumatology International Trials Organization criteria for JIA.^18^ The diagnosis of JIA was defined according to previously published algorithms^19^, ^20^, ^21^, ^22^ with the one or more of the following criteria:

1. Two or more JIA codes (Supplemental Tables 1 and 2) separated by ≥14 days and within 183 days.
2. One or more JIA codes by any provider plus one or more of the following:
  - Medication code for intra‐articular glucocorticoids injection only (Supplementary Table 3) in the 183 days following the JIA code,
  - Intra‐articular injection procedure code (Supplementary Table 3) in the 183 days following the JIA code, and
  - Claim for a disease‐modifying antirheumatic drug or biologic in the 183 days following the JIA code (Supplementary Table 4).
3. One or more codes indicating JIA by a rheumatologist.

To ensure cases represented incident diagnoses only, patients were excluded if they had less than one year of continuous enrollment before the index date or if they had a claim for a JIA diagnostic code during that period. The index date for patients was defined as the date of the first JIA code assignment. Age‐ and sex‐matched controls were identified at a 2:1 ratio within each insurance type (commercial or Medicaid). Healthy controls were excluded if (1) they did not have 12 months of continuous enrollment before their inclusion or (2) they had a diagnosis any of the complex chronic condition (CCCs) at cohort inclusion.^23^, ^24^ The index date for healthy controls was defined as the date of an outpatient visit occurring within two months of the index date of the matched JIA patient.

### Outcomes

The primary outcome was health care utilization as count data, which was defined as any inpatient admission, emergency department or urgent care (ED/UC) visit, or outpatient claim for a billing visit with any physician or advanced practice provider. Secondary outcomes included provider specialty type (generalist, orthopedist, rheumatologist, ophthalmologist, dermatologist, or other medical subspecialist) with specific analysis of orthopedic and generalist utilization for outpatient visits. Visits with a general practitioner were defined as any visit with a provider code from a pediatrician, family medicine provider, internal medicine provider, or general acute care provider as well as codes from medical doctors, nurse practitioners, and physicians’ assistants not elsewhere classified. ED/UC visits were defined as visits with a provider code from an emergency medicine provider or site code consistent with a care delivery in an ED/UC. Orthopedic visits were defined as any visit with a provider code from an orthopedist, a pediatric orthopedist, or a sports medicine physician.

### Covariates

Potential predictors for health care utilization included sex (male or female), age (continuous), and insurance type (commercial vs Medicaid). The geographic region was only available for those in the commercial insurance dataset and is categorized based on the patient's state of residence into the following categories: Northeast, Midwest, South, and West. Race and ethnicity (Black, Hispanic, White, other, or missing) were available for patients in the Medicaid database. Because these variables were not consistently available across groups, they were excluded from the primary adjusted models (which included patients from both the commercial and the Medicaid databases). There was no missingness of any of the covariates included in the primary adjusted models. All demographic factors were defined at time of the index or diagnosis date.

### Statistical analysis

All analyses were performed using R Studio version 12.0 (Posit). Descriptive statistics were used to summarize cohort characteristics and specialty utilization. Medians and interquartile ranges (IQRs) were calculated for total health care, outpatient, inpatient, and ED/UC visits, with limited medians also computed for patients with one or more visits to account for excess zeros.

Incidence rate ratios (IRRs) with 95% confidence intervals (CIs) were calculated to compare health care utilization between patients with JIA and healthy controls in the 12 months before the index date. For outcomes without zero inflation, Poisson and negative binomial (NB) models were considered, whereas for outcomes with excess zeros, hurdle models and zero‐inflated NB models were considered.^25^ Models were compared with Akaike's information criterion, Bayesian information criterion, and Vuong tests, and the best‐fitting, most parsimonious approach was selected. Ultimately, NB modeling was used for outcomes without zero inflation including total health care, outpatient, and generalist utilization, whereas hurdle modeling was used for outcomes with zero inflation including ED/UC, inpatient, and orthopedic utilization. Two types of modeling were used because outcomes with substantial zero inflation may reflect two distinct processes: the likelihood of any utilization of that type and the degree of utilization among those who access that type of care. For these outcomes, modeling that does not account for zero inflation would be challenging to interpret. Hurdle modeling is a two‐step process in which logistic regression is used to model a binary outcome of utilization versus no utilization (the decision to seek care), and, for those with some utilization, an NB regression is then performed to model visit counts among utilizers (degree of utilization). Odds ratios (ORs) were reported for the binary component, and IRRs for the count component in a hurdle model.^25^ All models were adjusted for sex, age at diagnosis, and insurance type. To better understand how utilization differed by insurance type, we conducted a subgroup analysis including only patients with JIA to compare the utilization between patients in the commercial versus Medicaid databases.

### Sensitivity analyses

**Figure 1:**
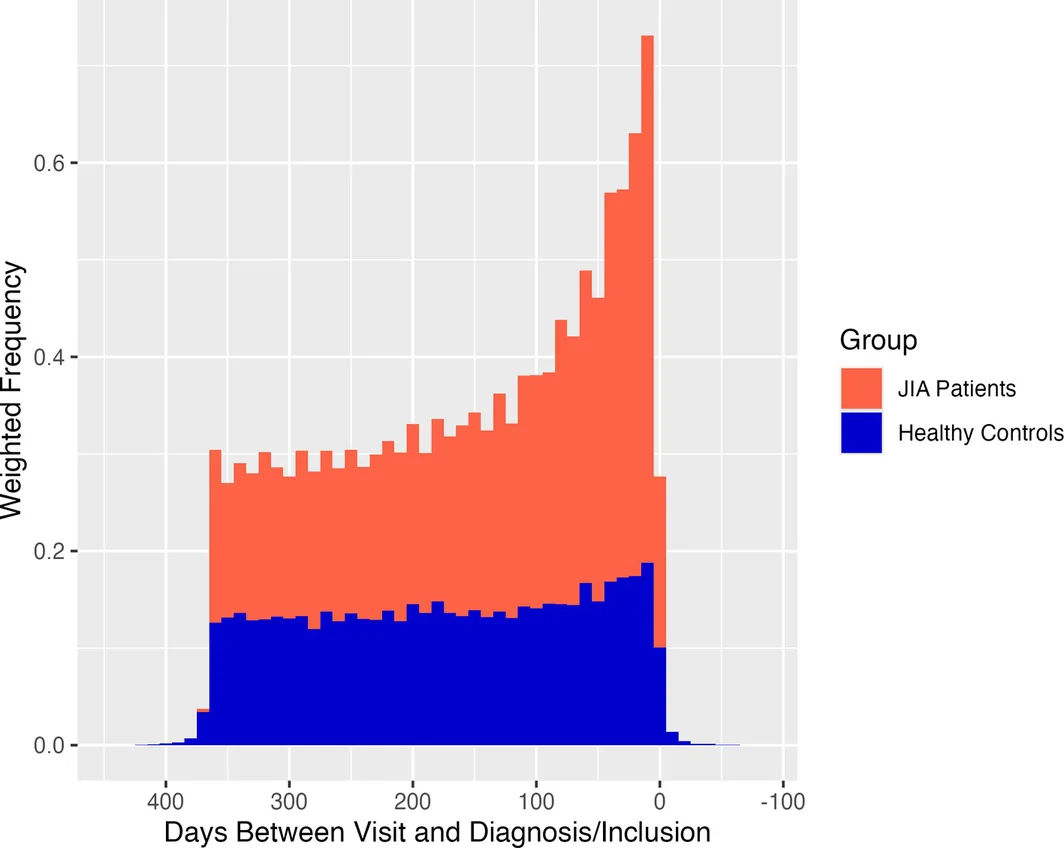
Time between visits and diagnosis/inclusion. This figure demonstrates the frequency of health care utilization in the outpatient, inpatient, or emergency department settings as patients approach the date of diagnosis (day 0) for patients with JIA or inclusion in the cohort for healthy controls. The visit frequencies are weighted to account for the number of patients versus controls. JIA, juvenile idiopathic arthritis.

To ensure utilization among patients with JIA was not driven up by preexisting chronic conditions, patients with any of the CCCs^23^, ^24^ were excluded from the analysis. The analyses were repeated using this adjusted cohort. Additionally, we examined the impact of race and ethnicity in the Medicaid cohort and geographic region in the commercial cohort. Finally, because multiple model types were used, IRR estimates were compared across model specifications to assess robustness. Because the study period included the COVID‐19 pandemic, an additional analysis was performed replicating Figure 1 for patients diagnosed in the prepandemic period (2014–2019), the pandemic period (2020–2021), and the postpandemic period (2022) (Supplemental Figure 1).

## RESULTS

### Cohort characteristics

We identified 10,021 children with an incident diagnosis of JIA and 20,042 age‐ and sex‐matched healthy controls. The cohort was 35.4% male with a median age at index date of 13 years (IQR 8–15). A majority of the population (59%) was commercially insured. Among patients with JIA, 93.7% had no CCCs before JIA diagnosis (Table 1).

**Table 1:** Cohort demographics*

	Patients with JIA (n = 10,021)	Healthy controls (n = 20,042)
Demographics		
Male, n (%)	3,552 (35.4)	7,104 (35.4)
Age at diagnosis, median (IQR)	13 (8–15)	13 (8–15)
Insurance type, n (%)		
Commercial	5,914 (59.0)	11,828 (59.0)
Medicaid	4,107 (41.0)	8,214 (41.0)
Geographic region of residence,^a^ n (%)		
Northeast	1,070 (18.1)	1,213 (10.3)
North central	1,380 (23.3)	2,676 (22.6)
South	2,275 (38.5)	7,093 (59.9)
West	1,131 (19.1)	830 (7.0)
Missing or unknown	58 (1.0)	16 (0.1)
Race,^b^ n (%)		
Black	792 (19.3)	2,714 (33.0)
Hispanic	317 (7.7)	756 (9.2)
White	2,431 (59.2)	3,942 (48.0)
Other	159 (3.9)	431 (5.3)
Missing	408 (9.9)	379 (4.6)
Number of complex chronic conditions, n (%)		
0	9,392 (93.7)	20,042 (100)
1	580 (5.8)	0 (0)
2+	49 (0.4)	0 (0)

* *International Classification of Diseases, Ninth Revision* (696.0, 714.x, 720.0, 720.81, 720.89, and 720.9) and *International Statistical Classification of Diseases and Related Health Problems, Tenth Revision* (M05.00, M06.xx, M08.xx, M45.x, M46.0x, M46.8x, M46.9x, and L40.5x) codes were used to determine cohort inclusion. IQR, interquartile range; JIA, juvenile idiopathic arthritis.

^a^ Region only available for patients with commercial insurance.

^b^ Race is only available for patients with Medicaid insurance.

### Overall health care utilization

Among the 10,021 children with JIA, there were 134,650 total visits composed of 118,103 outpatient provider visits, 15,384 ED/UC visits, and 1,163 inpatient hospitalizations in the year before diagnosis. Patients with JIA had a median of 10 total health care encounters (IQR 6–18), whereas controls had a median of four visits (IQR 2–8) (Table 2). Increased utilization was most pronounced in the outpatient setting in which patients with JIA had a median of nine visits (IQR 5–16) for patients with JIA compared with four visits (IQR 2–7) for healthy controls. Only 376 patients with JIA (3.8%) had no utilization preceding diagnosis, whereas 3,695 controls (18.4%) had no utilization. In the year preceding the index date, total health care utilization was significantly higher for patients with JIA versus healthy controls (IRR 2.55 [95% CI 2.49–2.62], *P* < 0.001), with increased utilization consistent across all settings after accounting for sex, age, and insurance type: outpatient (IRR 2.56 [95% CI 2.49–2.62], *P* < 0.001), inpatient (IRR 2.00 [95% CI 1.48–2.71], *P* < 0.001), and ED/UC encounters (IRR 1.76 [95% CI 1.67–1.86], *P* < 0.001) (Table 2). For zero‐inflated data, ORs of utilization versus no utilization were also calculated. Patients with JIA had significantly higher odds of inpatient utilization (OR 4.16 [95% CI 3.56–4.87]) and ED utilization (OR 2.36 [95% CI 2.24–2.49]) than the healthy controls. There was no statistically significant difference in total utilization among patients with JIA by age, insurance type, or sex. Interaction terms between case status and insurance status were added to the model to assess for potential effect modification. However, they were not statistically significant, and therefore, they were not included in the final parsimonious multivariable models.

**Table 2:** Health care utilization 12 months before index date*

	JIA cohort (n = 10,021), median^a^ (IQR) [range]	Healthy controls (n = 20,042), median^a^ (IQR) [range]	Odds ratio^b^ (95% CI)	Incidence rate ratio^c^ (95% CI)
Total health care utilization	10 (6–18) [0–376]	4 (2–8) [0–365]	–	2.55 (2.49–2.62)
Outpatient visits	9 (5–16) [0–376]	4 (2–7) [0–365]	–	2.56 (2.49–2.63)
ED/urgent care visits	2 (1–4) [0–69]	2 (1–3) [0–26]	2.36 (2.24–2.49)	1.76 (1.67–1.86)
Inpatient hospitalizations	1 (1–2) [0–18]	1 (1–1) [0–8]	4.16 (3.68–4.70)	2.00

* Visit numbers presented as median, IQR, and total range. Negative binomial models were utilized for total and outpatient health care utilization, and hurdle models were utilized for ED and inpatient utilization. CI, confidence interval; ED, emergency department; IQR, interquartile range; JIA, juvenile idiopathic arthritis.

^a^ Median and IQR calculated for only those patients with some health care utilization.

^b^ Odds ratios calculated for utilization vs no utilization in zero‐inflated outcomes.

^c^ Incidence rate ratios were adjusted for age, sex, and insurance type.

### Timing of utilization

Elevated levels of health care utilization among patients with JIA were observed from 12 months before JIA diagnosis, with an escalation in utilization starting around three months before diagnosis (Figure 1). This figure was replicated for the prepandemic, during‐pandemic, and postpandemic period (Supplementary Figure 1), which demonstrated that the utilization differences between patients with JIA and controls persisted despite the pandemic. All types of health care utilization became more frequent as the JIA diagnosis approached. Health care utilization remained stable over time in the healthy control population.

### Utilization by insurance type in patients with JIA

Although overall health care utilization did not differ between patients with JIA with Medicaid versus commercial insurance (IRR 1.03 [95% CI 0.99–1.07]), utilization patterns differed (Table 3). Patients with Medicaid coverage utilized ED care more frequently (OR 2.05 [95% CI 1.89–2.23]) and orthopedic care much less frequently (OR 0.27 [95% CI 0.24–0.34]). Utilization of outpatient providers was overall slightly lower (IRR 0.94 [95% CI 0.90–0.98]) in the population with Medicaid, but general practitioner utilization was higher (IRR 1.31 [95% CI 1.26–1.36]).

**Table 3:** Health care utilization for patients with JIA with Medicaid compared to those with commercial insurance*

	Odds ratio^a^ (95% CI)	Adjusted incidence rate ratio^b^ (95% CI)
Total health care utilization	–	1.03 (0.99–1.07)
Outpatient visits	–	0.94 (0.90–0.98)
General practitioner visits	–	1.31 (1.26–1.36)
Orthopedic visits	0.27 (0.24–0.34)	0.91 (0.78–1.06)
ED/urgent care visits	2.05 (1.89–2.23)	1.75 (1.62–1.88)
Inpatient hospitalizations	1.19 (1.03–1.38)	0.99 (0.731–1.36)

* Odds ratios and incidence rate ratios for health care utilization of patients with Medicaid vs those with commercial insurance. Negative binomial models were utilized for total, outpatient, and generalist health care utilization, and hurdle models were utilized for ED, inpatient, and orthopedic utilization. CI, confidence interval; ED, emergency department; JIA, juvenile idiopathic arthritis.

^a^ Odds ratios were calculated for utilization vs no utilization in zero‐inflated outcomes.

^b^ Incidence rate ratios were adjusted for age and sex.

### Utilization by specialty

JIA outpatient encounters were most frequent with generalists (84,507 visits; 71.6% of total outpatient visits) and orthopedists (5,743; 4.9%) in the prediagnosis period. Patients with JIA were also seen more frequently than healthy controls in the prediagnosis period by rheumatologists, ophthalmologists, dermatologists, and other medical subspecialists (Table 4). In the year preceding diagnosis, 91.8% (n = 9,203), 47.0% (n = 4,714), and 22.9% (n = 2,906) of children with JIA were seen by a general practitioner, ED/UC, or orthopedist, respectively.

**Table 4:** Frequency of visit to provider types in the year preceding diagnosis*

Provider type	Patient with JIA (visits per person‐year)	Healthy control (visits per person‐year)
General provider	8.4	3.52
Orthopedic provider	0.57	0.15
Rheumatology	0.31	<0.001
Ophthalmology	0.18	0.02
Dermatology	0.24	0.12
Other medical subspecialty	0.46	0.06

* Frequency of utilization by provider type in the year preceding diagnosis presented as visits per person‐year. JIA, juvenile idiopathic arthritis.

### Sensitivity analyses

In a sensitivity analysis that removed patients with CCCs from the JIA cohort (n = 629), JIA cases continued to have excess health care utilization across all settings in the year preceding diagnosis when compared to controls. The OR and IRR of inpatient utilization decreased from 4.16 (95% CI 3.56–4.87) and 2.00 (95% CI 1.48–2.71) to 3.42 (95% CI 3.03–3.91) and 1.89 (95% CI 1.38–2.60), respectively, with the exclusion of patients with CCCs, but this outcome remains statistically significant. In subgroup analyses, inclusion of geographic region in models restricted to commercially insured patients and inclusion of race in models restricted to Medzicaid patients did not substantially alter the observed differences in health care utilization between patients with JIA and controls.

## DISCUSSION

This study quantifies and describes health care utilization in a large, population‐based cohort of US patients with an incident diagnosis of JIA and demonstrates that patients with JIA consistently utilize more health care in the year preceding diagnosis when compared to healthy controls, across all health care settings. Utilization among these patients is elevated from the time of cohort inclusion and escalates closer to the time of diagnosis, demonstrating that many patients are frequently seeking care for their symptoms, for at least a year before JIA diagnosis. In this cohort, patients sought care from generalists, orthopedists, and ED/UC centers before diagnosis. Interestingly, patients insured by Medicaid were more likely to seek emergency care, whereas commercially insured patients were more likely to seek orthopedic care. A better understanding of these trajectories of health care utilization preceding JIA diagnosis is a critical first step to designing interventions to improve time to diagnosis and targeted treatment.

Several findings from this study are worth exploring further. Surprisingly, patients with JIA in this cohort had more than a two‐fold higher level of health care utilization from time of cohort inclusion. In designing the current study, the duration of one year preceding diagnosis was selected based on prior data that suggested a median time to diagnosis of three to four months for patients with JIA.^26^, ^27^ However, the utilization patterns in the cohort demonstrated that patients have almost twice the utilization of healthy controls a year before diagnosis, which suggests that the diagnostic process may start earlier and take longer than prior studies have suggested. Although a stepwise diagnostic evaluation for a cause of joint pain may be expected to take weeks to months, as reflected in the acute rise in health care utilization of patients with JIA just before diagnosis, it is unlikely to explain high health care utilization spanning a year. This utilization suggests that there are multiple visits with health care professionals at which an earlier JIA diagnosis may be possible or that there are subtle early symptoms of JIA that are being missed in the clinical setting. This is consistent with prior qualitative studies of patients with JIA that have characterized the diagnostic journey as “turbulent,” requiring multiple interactions with the health care system and significant parental persistence for a diagnosis to ultimately be made.^28^, ^29^ High health care utilization before diagnosis has also been documented in other pediatric rheumatic diseases including systemic lupus erythematosus^30^ and inflammatory bowel disease (IBD).^31^ In pediatric IBD, elevated levels of health care utilization were noted for up to four years before diagnosis, implying the existence of a preclinical symptomatic period or of prolonged diagnostic delay.^31^ Studies of time to diagnosis in adult inflammatory arthritis have yielded results with diagnostic delays lasting up to a decade in some patients.^32^, ^33^ It is important to note that, although the diagnostic intervals presented within for children with JIA are shorter than those for equivalent adult conditions, active arthritis in childhood comes with unique risks for acquired deformities during periods of active growth, such as leg length discrepancy and negative developmental effects.^1^

Although this study does not elucidate the reasons for diagnostic delay in JIA, many factors make JIA a diagnostically complex condition, including its subtle and heterogenous clinical presentation, the overlap of symptoms with more common pediatric conditions, and the lack of a single, true diagnostic test. As such, the initial clinicians interacting with these patients may be poorly equipped to recognize it, and the development of screening tools and management guidance for these providers may be necessary to accelerate appropriate referral. The workforce shortage and geographic limitation of the pediatric rheumatology workforce in the United States^34^, ^35^ may contribute to diagnostic delays and heightens the importance that nonrheumatologic providers can recognize and appropriately refer patients with inflammatory arthritis. In developing interventions to improve the diagnostic process, it is important to understand the settings in which patients are receiving care. In the presented cohort, utilization was most frequent with general practitioners, orthopedists, and ED/UC providers. The vast majority of patients with JIA (91.8%) were seen by a general practitioner in the year preceding diagnosis, nearly half (47.0%) were seen in an ED/UC, and 22.9% were seen by orthopedists. These clinical settings may be the highest yield targets for interventions to encourage early referral for pediatric rheumatology assessment in appropriate patients.

Beyond general patterns of utilization, our analysis also highlights differential health care utilization between patients in the commercial and Medicaid databases. There was increased utilization of ED/UC centers and lower utilization of orthopedic care among patients in the Medicaid‐insured database. This is consistent with data from studies across multiple disease processes that demonstrate that Medicaid patients seek more care from the ED and less from subspecialists.^36^, ^37^, ^38^ With MarketScan data, we are unable to determine the etiology of these differential utilization patterns between insurance types and whether other demographic factors, such as socioeconomic status, geographic region, or race and ethnicity, are the drivers of the differences. Although this differential utilization is likely multifactorial, the pattern makes it clear that multiple, setting‐specific interventions will be needed to improve diagnosis equitably for children with all insurance types.

The strengths of this study include the use of a national administrative database, which has generated a large cohort and enabled the capture of interactions across multiple health care systems. Capturing data across health systems is particularly important in studies related to diagnostic delay/error because prior studies have demonstrated that patients are more likely to seek care across systems when an error occurs or a diagnosis is not received.^39^ This study also has several limitations. Although utilizing both the commercial and Medicaid databases improves the generalizability of the presented study, it is important to note that geographic location is only available for patients with commercial insurance and race and ethnicity data are only available for those with Medicaid insurance, which limited the subgroup analyses that could be performed based on demographic factors. However, a sensitivity analysis was performed to ensure that correction for these factors did not alter the point estimate or statistical significance of differences between cases and controls. As with any administrative database study, misclassification is also possible, and there is no validated method for identifying incident cases of JIA. By leveraging previously published JIA identification algorithms,^19^, ^20^, ^21^, ^22^ we aimed to minimize any misclassification.

In summary, this study leveraged administrative data to characterize the frequency, patterns, and types of health care utilization before diagnosis of JIA, which is essential to identifying system issues and practice patterns preceding JIA diagnosis. This study demonstrated that patients with JIA utilize significantly more health care in the year preceding diagnosis, particularly from generalists, orthopedists, and emergency care providers, and that this high utilization starts at least a year before diagnosis. These utilization patterns indicate that the diagnostic journey for patients with JIA may be more prolonged and nuanced than previously believed and that there may be opportunities for earlier recognition of key symptoms and referral to rheumatology care.

## AUTHOR CONTRIBUTIONS

All authors contributed to at least one of the following manuscript preparation roles: conceptualization AND/OR methodology, software, investigation, formal analysis, data curation, visualization, and validation AND drafting or reviewing/editing the final draft. As corresponding author, Dr Costello confirms that all authors have provided the final approval of the version to be published and takes responsibility for the affirmations regarding article submission (eg, not under consideration by another journal), the integrity of the data presented, and the statements regarding compliance with institutional review board/Declaration of Helsinki requirements.

## Supporting information


**Disclosure Form**:

**Data S1** Supporting Information

## References

[1] Petty RE , Laxer RM , Lindsley CB , et al. Textbook of pediatric rheumatology. 8th ed. Elsevier; 2020.

[2] Shishov M , Weiss PF , Levy DM , et al; Pediatric Rheumatology Collaborative Study Group Advisory Council. 25 Years of biologics for the treatment of pediatric rheumatic disease: advances in prognosis and ongoing challenges. Arthritis Care Res (Hoboken) 2025;77(5):573–583. doi:10.1002/acr.25482 PMC12915560 39651592

[3] de Jonge JB , de Roock S , Schonenberg‐Meinema D , et al; UCAN CAN‐DU and UCAN CURE consortia . Effect of time to start of biologic therapy on treatment response in childhood arthritis: results from the UCAN CAN‐DU study. *Arthritis* Rheumatol 2026;78(3):743–751. doi:10.1002/art.43401 PMC12991923 40977426

[4] Ravichandran N , Guleria S , Mohindra N , et al. Predictors of long‐term functional outcomes of juvenile idiopathic arthritis‐enthesitis‐related arthritis: a single centre experience. Rheumatology (Oxford) 2023;62(9):3110–3116. doi:10.1093/rheumatology/kead032 36702467

[5] Wallace CA , Giannini EH , Spalding SJ , et al; Childhood Arthritis and Rheumatology Research Alliance (CARRA). Clinically inactive disease in a cohort of children with new‐onset polyarticular juvenile idiopathic arthritis treated with early aggressive therapy: time to achievement, total duration, and predictors. J Rheumatol 2014;41(6):1163–1170. doi:10.3899/jrheum.131503 24786928

[6] Wallace CA , Giannini EH , Spalding SJ , et al; Childhood Arthritis and Rheumatology Research Alliance. Trial of early aggressive therapy in polyarticular juvenile idiopathic arthritis. Arthritis Rheum 2012;64(6):2012–2021. doi:10.1002/art.34343 PMC3319524 22183975

[7] Minden K , Horneff G , Niewerth M , et al. Time of disease‐modifying antirheumatic drug start in juvenile idiopathic arthritis and the likelihood of a drug‐free remission in young adulthood. Arthritis Care Res (Hoboken) 2019;71(4):471–481. doi:10.1002/acr.23709 30044538

[8] Costello A , Muir C , Rui X , et al. Delayed diagnosis and accrual of joint damage in incident cases of juvenile idiopathic arthritis [abstract]. Arthritis Rheumatol 2024; 76(suppl 9). https://acrabstracts.org/abstract/delayed-diagnosis-and-accrual-of-joint-damage-in-incident-cases-of-juvenile-idiopathic-arthritis/.

[9] Shiff NJ , Tucker LB , Guzman J , et al. Factors associated with a longer time to access pediatric rheumatologists in Canadian children with juvenile idiopathic arthritis. J Rheumatol 2010;37(11):2415–2421. doi:10.3899/jrheum.100083 20716664

[10] Barber CEH , Barnabe C , Benseler S , et al. Patient factors associated with waiting time to pediatric rheumatologist consultation for patients with juvenile idiopathic arthritis. Pediatr Rheumatol Online J 2020;18(1):22. doi:10.1186/s12969-020-0413-7 PMC7059295 32143720

[11] Boiu S , Marniga E , Bader‐Meunier B , et al. Functional status in severe juvenile idiopathic arthritis in the biologic treatment era: an assessment in a French paediatric rheumatology referral centre. Rheumatology (Oxford) 2012;51(7):1285–1292. doi:10.1093/rheumatology/kes004 22389127

[12] Chausset A , Lambert C , Belot A , et al. Individual and environmental determinants associated with longer times to access pediatric rheumatology centers for patients with juvenile idiopathic arthritis, a JIR cohort study. Pediatr Rheumatol Online J 2023;21(1):24. doi:10.1186/s12969-023-00809-8 PMC10015663 36918902

[13] Marino A , Baldassarre P , Ferrigno C , et al. Pre‐rheumatology referral consultation and investigation pattern in children with joint complaints: focus on juvenile idiopathic arthritis. Children (Basel) 2024;11(5):600. doi:10.3390/children11050600 PMC11120367 38790595

[14] Verstappen SMM , Cobb J , Foster HE , et al. The association between low socioeconomic status with high physical limitations and low illness self‐perception in patients with juvenile idiopathic arthritis: results from the Childhood Arthritis Prospective Study. Arthritis Care Res (Hoboken) 2015;67(3):382–389. doi:10.1002/acr.22466 PMC4737227 25187470

[15] McErlane F , Foster HE , Carrasco R , et al. Trends in paediatric rheumatology referral times and disease activity indices over a ten‐year period among children and young people with juvenile idiopathic arthritis: results from the childhood arthritis prospective study. Rheumatology (Oxford) 2016;55(7):1225–1234. doi:10.1093/rheumatology/kew021 PMC4911538 27016664

[16] Foster HE , Eltringham MS , Kay LJ , et al. Delay in access to appropriate care for children presenting with musculoskeletal symptoms and ultimately diagnosed with juvenile idiopathic arthritis. Arthritis Rheum 2007;57(6):921–927. doi:10.1002/art.22882 17665486

[17] Merative . MarketScan research databases for life sciences researchers. Merative; 2023. Accessed October 10 2024. https://www.merative.com/documents/marketscan‐for‐life‐sciences‐researchers

[18] Martini A , Ravelli A , Avcin T , et al; Pediatric Rheumatology International Trials Organization (PRINTO). Toward new classification criteria for juvenile idiopathic arthritis: first steps, Pediatric Rheumatology International Trials Organization international consensus. J Rheumatol 2019;46(2):190–197. doi:10.3899/jrheum.180168 30275259

[19] Buckley LH , Xiao R , Perman MJ , et al. Psoriasis associated with tumor necrosis factor inhibitors in children with inflammatory diseases. Arthritis Care Res (Hoboken) 2021;73(2):215–220. doi:10.1002/acr.24100 PMC8176628 31646743

[20] Beukelman T , Haynes K , Curtis JR , et al; Safety Assessment of Biological Therapeutics Collaboration. Rates of malignancy associated with juvenile idiopathic arthritis and its treatment. Arthritis Rheum 2012;64(4):1263–1271. doi:10.1002/art.34348 PMC3315602 22328538

[21] Bilgic Dagci AO , Chang JC , Xiao R , et al. Opioid use in children with inflammatory bowel disease‐related arthritis. Clin Exp Rheumatol 2023;41(7):1553–1560. doi:10.55563/clinexprheumatol/3bu1sf PMC10523932 37083174

[22] Brandon TG , Manos CK , Xiao R , et al. Pediatric psoriatic arthritis: a population‐based cohort study of risk factors for onset and subsequent risk of inflammatory comorbidities. J Psoriasis Psoriatic Arthritis 2018;3(4):131–136. doi:10.1177/2475530318799072 PMC6660180 31355354

[23] Feinstein JA , Hall M , Davidson A , et al. Pediatric complex chronic condition system version 3. JAMA Netw Open 2024;7(7):e2420579. doi:10.1001/jamanetworkopen.2024.20579 PMC11250371 39008301

[24] Feudtner C , Feinstein JA , Zhong W , et al. Pediatric complex chronic conditions classification system version 2: updated for ICD‐10 and complex medical technology dependence and transplantation. BMC Pediatr 2014;14(1):199. doi:10.1186/1471-2431-14-199 PMC4134331 25102958

[25] Feng CX . A comparison of zero‐inflated and hurdle models for modeling zero‐inflated count data. J Stat Distrib Appl 2021;8(1):8. doi:10.1186/s40488-021-00121-4 PMC8570364 34760432

[26] Chausset A , Pereira B , Echaubard S , et al. Access to paediatric rheumatology care in juvenile idiopathic arthritis: what do we know? A systematic review. Rheumatology (Oxford) 2020;59(12):3633–3644. doi:10.1093/rheumatology/keaa438 32940701

[27] Costello A , Rasooly I , Weiss P . Rheum for improvement? Delayed diagnosis of juvenile idiopathic arthritis: a narrative review. Arthritis Care Res (Hoboken) 2025;77(3):283–290. doi:10.1002/acr.25438 PMC11848998 39308000

[28] Rapley T , May C , Smith N , et al. ‘Snakes & ladders’: factors influencing access to appropriate care for children and young people with suspected juvenile idiopathic arthritis ‐ a qualitative study. Pediatr Rheumatol Online J 2021;19(1):43. doi:10.1186/s12969-021-00531-3 PMC7986503 33757545

[29] Chausset A , Freychet C , Lohse A , et al. Diagnosis journey for children with juvenile idiopathic arthritis: a qualitative study. Arch Dis Child. 2024;109(12):1003–1009. doi:10.1136/archdischild-2024-327426 39174297

[30] Chang JC , Mandell DS , Knight AM . High health care utilization preceding diagnosis of systemic lupus erythematosus in youth. Arthritis Care Res (Hoboken) 2018;70(9):1303–1311. doi:10.1002/acr.23485 PMC5984118 29195017

[31] Singh H , Nugent Z , Targownik LE , et al. Health care use by a population‐based cohort of children with inflammatory bowel disease. Clin Gastroenterol Hepatol 2015;13(7):1302–1309.e3. doi:10.1016/j.cgh.2015.01.022 25638585

[32] Ogdie A , Rozycki M , Arndt T , et al. Longitudinal analysis of the patient pathways to diagnosis of psoriatic arthritis. Arthritis Res Ther 2021;23(1):252. doi:10.1186/s13075-021-02628-2 PMC8485539 34598717

[33] Hay CA , Packham J , Ryan S , et al. Diagnostic delay in axial spondyloarthritis: a systematic review. Clin Rheumatol 2022;41(7):1939–1950. doi:10.1007/s10067-022-06100-7 PMC9187558 35182270

[34] Correll CK , Ditmyer MM , Mehta J , et al. 2015 American College of Rheumatology workforce study and demand projections of pediatric rheumatology workforce, 2015–2030. Arthritis Care Res (Hoboken) 2022;74(3):340–348. doi:10.1002/acr.24497 33107674

[35] Correll CK , Klein‐Gitelman MS , Henrickson M , et al. Child health needs and the pediatric rheumatology workforce: 2020–2040. Pediatrics 2024;153(suppl 2):e2023063678R. doi:10.1542/peds.2023-063678R 38300008

[36] Kim H , McConnell KJ , Sun BC . Comparing emergency department use among Medicaid and commercial patients using all‐payer all‐claims data. Popul Health Manag 2017;20(4):271–277. doi:10.1089/pop.2016.0075 PMC5564052 28075692

[37] Hsiang WR , Lukasiewicz A , Gentry M , et al. Medicaid patients have greater difficulty scheduling health care appointments compared with private insurance patients: a meta‐analysis. Inquiry 2019;56:0046958019838118. doi:10.1177/0046958019838118 PMC6452575 30947608

[38] Bisgaier J , Rhodes KV . Auditing access to specialty care for children with public insurance. N Engl J Med 2011;364(24):2324–2333. doi:10.1056/NEJMsa1013285 21675891

[39] Vaillancourt S , Guttmann A , Li Q , et al. Repeated emergency department visits among children admitted with meningitis or septicemia: a population‐based study. Ann Emerg Med 2015;65(6):625–632.e3. doi:10.1016/j.annemergmed.2014.10.022 25458981

