# Supplementary material for: High Health Care Utilization Preceding Diagnosis With Juvenile Idiopathic Arthritis
Source: Arthritis Care Res (Hoboken). 2026 Apr 22;78(10):1544–51. doi: 10.1002/acr.80037 (PMC13613251; doi:10.1002/acr.80037)
Supplement: Supplementary file 2 [file ACR-78-1544-s002.docx]

| **Supplemental Table 1: ICD-9 and 10 Codes Utilized in Data Definition** | | | |
| --- | --- | --- | --- |
| **ICD-9 code** | **Description** | **ICD-10 Code** | **Description** |
| 714.x | Rheumatoid arthritis and other inflammatory polyarthropathies | M05.xx | Seropositive rheumatoid arthritis |
|  |  | M06.xx | Other rheumatoid arthritis |
|  |  | M08.xx | Juvenile arthritis |
| 720.xx | Ankylosing spondylitis and other inflammatory spondylopathies | M45.xx | Ankylosing spondylitis |
|  |  | M46.0x | Spinal enthesopathy |
|  |  | M46.1 | Sacroiliitis, not elsewhere classified |
|  |  | M46.8x | Other specified inflammatory spondylopathies. |
|  |  | M46.9x | Unspecified inflammatory spondylopathy. |
| 696.0 | Psoriatic arthropathy | L40.5x | Arthropathic psoriasis |
| **Legend.** International Classification of Disease Codes utilized in data definition of cases of JIA. Codes 720.1, 720.2, M46.1, and M46.0 (non-specific sacroiilitis codes) were dropped unless the patient was prescribed a biologic or a joint injection. Patients were additionally excluded if they received their JIA ICD-9/10 codes from a provider who cannot diagnose or manage the condition (Supplemental Table 2) and never received a biologic or joint injection. ICD, International Classification of Diseases | | | |

| **Supplemental Table 2: Provider Types Excluded from Being the Diagnosing Provider in Case Definition** | |
| --- | --- |
| **STDPROV Code** | **Provider description** |
| 20 | Mental Health/Chemical Dep NEC |
| 21 | Mental Health Facilities |
| 22 | Chemical Depend Treatment Ctr |
| 38 | Hospice Facility |
| 120 | Chiropractor/DCM |
| 130 | Podiatry |
| 160 | Nuclear Medicine |
| 815 | Medical Technician |
| 822 | Nursing Services |
| 850 | Therapy (Physical) |
| 853 | Therapists (Supportive) |
| 855 | Therapists (Alternative) |
| 857 | Renal Dialysis Therapy |
| 860 | Psychologist |
| 865 | Acupuncturist |
| 900 | Health Educator/Agency |
| 905 | Transportation |
| 920 | Home Health Organiz/Agency |
| 925 | Imaging Center |
| 930 | Laboratory |
| 940 | Supply Center |
| 950 | Public Health Agency |
| Legend. The above provider types were not eligible to be the diagnosing provider of JIA per the case definition. To enhance our cohorts accuracy, we wanted a stringent definition of JIA cases. Therefore, diagnoses from these are provider types that cannot manage or treat JIA independently without a supervising physician or advanced practice provider were not allowed in the case definition. | |

| **Supplementary Table 3: Intraarticular Corticosteroid Injections** | |
| --- | --- |
| Generic Medications for which all Intra-articular National Drug Codes were included | Current Procedural Terminology Codes for Joint Injections |
| Triamcinolone acetonide | 20600 |
| Triamcinolone hexacetonide | 20604 |
| *Methylprednisolone acetate* | 20605 |
| Methylprednisonolone sodium succincate | 20606 |
| Triamcinolone diacetate | 20610 |
| Dexamethasone acetate | 20611 |
| Dexamethasone Sodium Phosphate |  |
| Hydrocortisone acetate |  |
| Hydrocortisone sodium phosphate |  |
| Hydrocortisone sodium succinate |  |
| Betamethasone ace/betamethasone Na Phos |  |
| Betamethasone sodium phosphate |  |
| Legend. Receipt of a joint injection was one of the criteria that was used to meet the JIA case definition. Receiving a joint injection was defined as either an insurance claim for any the intrarticular formulations of the above generic medications or an insurance claim for any of the above procedure codes for joint injection. JIA, Juvenile Idiopathic Arthritis | |

**Supplementary Table 4: Generic DMARD or Biologic Medication Names Utilized in Case Definition Criteria**

Methotrexate

Hydroxychloroquine

Leflunomide

Sulfasalazine

Mycophenolate

Infliximab

Etanercept

Adalimumab

Certolizumab

Golimumab

Abatacept

Abatacept

Canakinumab

Toclizumab

Tofacitinib

Rituximab

Secukinumab

Ustekinumab

**Legend.** An insurance claim for any of the above disease modifying anti-rheumatic drugs and biologics were utilized in the case definition criteria. All biosimilars and formulations were included.

Supplemental Figure 1:

| **Supplemental Figure 1:** Healthcare Utilization Patterns in Patients with JIA versus Healthy Controls Before, During, and After the SARS-CoV2 Pandemic | | |
| --- | --- | --- |
|  |  |  |
| **Legend.** This figure demonstrates the frequency of healthcare utilization in the outpatient, inpatient, and ED settings as patients approach the date of diagnosis (Day 0) for patients with JIA or inclusion in the cohort for healthy controls. The visit frequencies are weighted to account for the numbers of patients versus controls. As the SAR-CoV2 pandemic occurred during the study period, this figure demonstrates the consistency of utilization patterns pre-pandemic (Panel A, 2014-2019), during the pandemic (Panel B: 2020-2021), and post-pandemic (2022). | | |
